# Spatial aspects of oncogenic signalling determine the response to combination therapy in slice explants from Kras‐driven lung tumours

**DOI:** 10.1002/path.5059

**Published:** 2018-04-02

**Authors:** Katja Närhi, Ashwini S Nagaraj, Elina Parri, Riku Turkki, Petra W van Duijn, Annabrita Hemmes, Jenni Lahtela, Virva Uotinen, Mikko I Mäyränpää, Kaisa Salmenkivi, Jari Räsänen, Nina Linder, Jan Trapman, Antti Rannikko, Olli Kallioniemi, Taija M Af Hällström, Johan Lundin, Wolfgang Sommergruber, Simon Anders, Emmy W Verschuren

**Affiliations:** ^1^ Institute for Molecular Medicine Finland (FIMM), HiLIFE University of Helsinki Helsinki Finland; ^2^ Department of Urology, Josephine Nefkens Institute Erasmus Medical Center Rotterdam The Netherlands; ^3^ Department of Pathology University of Helsinki Helsinki Finland; ^4^ HUSLAB, Division of Pathology Helsinki University Hospital Helsinki Finland; ^5^ Heart and Lung Centre, Department of General Thoracic and Oesophageal Surgery Helsinki University Hospital Helsinki Finland; ^6^ Department of Pathology, Josephine Nefkens Institute Erasmus Medical Centre Rotterdam The Netherlands; ^7^ Department of Urology Helsinki University Hospital Helsinki Finland; ^8^ Orion Corporation Orion Pharma Espoo Finland; ^9^ Department of Lead Discovery Boehringer Ingelheim RCV GmbH & Co. KG Vienna Austria; ^10^ Centre for Molecular Biology of the University of Heidelberg (ZMBH) Heidelberg Germany

**Keywords:** non‐small‐cell lung cancer, prostate cancer, oncogenic signalling, precision‐cut slices, targeted therapy, spatial heterogeneity

## Abstract

A key question in precision medicine is how functional heterogeneity in solid tumours informs therapeutic sensitivity. We demonstrate that spatial characteristics of oncogenic signalling and therapy response can be modelled in precision‐cut slices from Kras‐driven non‐small‐cell lung cancer with varying histopathologies. Unexpectedly, profiling of in situ tumours demonstrated that signalling stratifies mostly according to histopathology, showing enhanced AKT and SRC activity in adenosquamous carcinoma, and mitogen‐activated protein kinase (MAPK) activity in adenocarcinoma. In addition, high intertumour and intratumour variability was detected, particularly of MAPK and mammalian target of rapamycin (mTOR) complex 1 activity. Using short‐term treatment of slice explants, we showed that cytotoxic responses to combination MAPK and phosphoinositide 3‐kinase–mTOR inhibition correlate with the spatially defined activities of both pathways. Thus, whereas genetic drivers determine histopathology spectra, histopathology‐associated and spatially variable signalling activities determine drug sensitivity. Our study is in support of spatial aspects of signalling heterogeneity being considered in clinical diagnostic settings, particularly to guide the selection of drug combinations. © 2018 The Authors. *The Journal of Pathology published* by John Wiley & Sons Ltd on behalf of Pathological Society of Great Britain and Ireland.

## Introduction

Cancer biomedicine is experiencing a surge in the approval of compounds that target driver mutations, and targetable mutations are particularly prevalent in non‐small‐cell lung cancer (NSCLC) 1. However, substantial genomic and functional tumour heterogeneity compromises treatment efficacy 2, and this includes heterogeneity in oncogenic signalling activities 3. Despite the availability of a range of molecules to target signalling, remarkably little is known about the *in situ* heterogeneity of signalling activities, and their context dependence.

When the response to targeted drugs is studied *ex vivo*, it is important for disease models to reflect the tumour‐intrinsic heterogeneity in oncogenic functions, including signalling. Precision‐cut tumour slices, which capture the native tumour in its microenvironment, constitute an attractive model with which to address this need. Within the IMI‐PREDECT project (http://www.predect.eu), we developed a workflow for short‐term culture of tumour slices, and showed that organotypic supports and atmospheric oxygen are strict requirements for tissue viability 4. A number of other studies have shown that responses to cytotoxic agents or selected targeted compounds can be modelled in clinical tumour‐derived slices 5, 6, 7, 8, 9, 10, 11. However, the question remains of how the *in situ* spatial signalling heterogeneity affects the pharmacodynamics of signalling inhibitors.

We set out to answer these questions by using two widely studied murine NSCLC models, namely those driven by the *Kras* ^*G12D*^ (*Kras*) oncogene, concomitantly with loss of either the tumour suppressor gene *Trp53* (*p53*) 12 or the serine/threonine kinase 11 gene (*Stk11*, also known as *Lkb1*) 13, 14, 15, 16. These are the most common drivers of clinical NSCLC, with approximate gene alteration rates of 20% for *LKB1*, 36% for *KRAS* and 46% for *TP53* in adenocarcinomas (ACs) 17, 18. Tumours are initiated from different progenitors, using adenoviruses that predominantly target Cre recombinase to either alveolar type II cells expressing surfactant protein C (Ad5‐SPC‐Cre), or to bronchiolar club cells expressing club cell antigen 10 (Ad5‐CC10‐Cre) 19, 20. *Kras;p53* (KP) 21 tumours initiated by either of these viruses lead to the formation of ACs and papillary ACs of varying grades 19. In contrast, *Kras;Lkb1* (KL) mice develop more aggressive tumours with an expanded histopathology spectrum 14, 16, and we recently showed that histotype spectra are dependent on the cell of origin: whereas alveolar progenitors predominantly lead to the formation of papillary AC, bronchiolar progenitors predominantly lead to the formation of adenosquamous carcinoma (ASC) and occasional mucinous or acinar AC 22. Importantly, we found that KL‐driven papillary AC and ASC histotypes show differences in immune‐related gene signatures and immune microenvironments 22.

To assess the impact of spatial signalling heterogeneity on therapeutic sensitivities, we first profiled *Kras*‐associated signalling activities, and proliferation and viability markers, in KP and KL NSCLC lesions. Our data show that, whereas genetic drivers define the histopathology spectrum, tumour‐specific signalling mostly aligns with histopathology, but also shows high intralesion and interlesion heterogeneity. Importantly, cytotoxic drug responses to *ex vivo* treatment with *Kras*‐related signalling inhibitors correspond with regional activities of the targeted pathways. Our study thus emphasises the need, and informs on methods, to consider the full complexity of spatial oncogenic signalling in diagnostic assay design.

## Materials and methods

### Mice and tissue preparation

Animals, breeding and intranasal infections with progenitor cell‐directed Ad5‐Cre viruses to initiate tumour formation were performed as described previously 22. Moribund mice were killed by cervical dislocation, and tumour‐bearing lungs were either processed for tumour slice culture, or immediately fixed with 4% formaldehyde overnight at ambient temperature. Precision‐cut tumour slices were fixed with 4% formaldehyde overnight at 4 °C. Fixed samples were paraffin‐embedded, and sections (4 μm) were processed for haematoxylin and eosin (H&E) staining or immunohistochemical (IHC) analysis. Animal handling and studies were performed according to guidelines from the Finnish National Board of Animal Experimentation (ESAVI/857/04.10.07/2013).

### Human lung cancer specimens and tissue microarrays (TMAs)

Surgically resected tumour specimens were received from NSCLC patients, with informed consent, at the Hospital District of Helsinki and Uusimaa (HUS), as approved by the ethical board of the Joint Authority for the HUS, Finland (Dnro: 85/13/03/00/15). Surgical specimens were dissected by a clinical pathologist prior to slicing. For TMAs, archived formalin‐fixed paraffin‐embedded tumour specimens were collected from 66 NSCLC patients operated on during 2000–2015 at the Hospital District of Helsinki. According to the International Association for the Study of Lung Cancer/American Thoracic Society/European Respiratory Society NSCLC classification system 23, 13 specimens were diagnosed as ASC, 25 as papillary AC, and 28 as squamous cell carcinoma (SCC). TMAs were prepared manually with 2‐mm‐diameter cores. Depending on tumour size, two or three replicate cores were included, and both SCC and AC components of ASC tumours were represented.

### Tumour slicing, culture, and drug treatments

Murine NSCLC tumours and clinical specimens were precision‐cut to make 200‐μm slices with a VT1200S microtome (Leica Biosystems, Wetzlar, Germany), as described previously 4. Murine slices were transferred to rotating incubators (Alabama Research & Development, Munford, AL, USA) (supplementary material, Figure S2A), within 90–120 min after mice had been killed. Surgical specimens arrived for slicing within 3 h postsurgery; slices were placed in culture within the next 60–90 min. Each day, 80% of the medium (F‐12; Thermo Fisher, Waltham, MA, USA) was replenished with fresh medium. Selumetinib (sel) (AZD624; Selleckchem, Munich, Germany), dactolisib (dact) (NVP‐BEZ235; Selleckchem) and saracatinib (sar) (LC Laboratories, Woburn, MA, USA) were stored at –80 °C in dimethyl sulphoxide (DMSO), and diluted with serum‐free culture medium to 0.5 μm, 0.5 or 1 μm, or 1 μm, respectively. Slices were fixed and processed at set time points. Relative changes in marker expression or dead cells were determined by comparing neighbouring DMSO‐treated or drug‐treated cultured slices with neighbouring uncultured (0 h) slices. More details on culture conditions and methods are provided in supplementary material, Supplementary methods Table S1.

### IHC and biomarker analysis

Standard IHC analysis was performed with dewaxed and rehydrated paraffin sections as described previously 4, with rabbit primary antibodies (supplementary material, Supplementary methods Table S2) and BrightVision poly‐horseradish peroxidase goat anti‐rabbit and 3,3′‐diaminobenzidine for detection (Immunologic, Duiven, The Netherlands). Dehydrated stained sections were counterstained with haematoxylin. Whole slide image scans (Pannoramic 250 scanner; 3DHISTECH) were used to quantify marker expression area (%; cytoplasmic, nuclear, or membranous) in manually selected regions of interest, by use of the Definiens Tissue Studio software (Definiens, Munich, Germany). For quantitative IHC analysis of marker expression areas in uncultured tissue slices, Adobe Photoshop CS6 (Adobe Systems, San Jose, CA, USA) was used to draw masks, and MATLAB (MathWorks, Natick, MA, USA) was used to define viable tumour areas.

### Quantification of necrosis and IHC results

H&E‐stained sections of tumour slices were pre‐analysed by a pathologist prior to masks being drawn on necrotic tumour areas with Adobe Photoshop CS6, as described previously 4. Relative decreases in viability were calculated as follows: (1 – viability in drug‐treated/viability in DMSO control) × 100%. Quantification of the phosphoprotein expression areas was performed with Photoshop CS6, by drawing masks on the stained areas; drawn masks were overlapped, and the overlapping area was recoloured in green with the magic wand tool in Photoshop CS6; the resulting differentially coloured areas of single and overlapping stains were measured with MATLAB or with Photoshop's histogram tool, and normalised to the total tumour area.

### Statistical analysis

Data visualisation and statistical analyses were performed with RStudio (RStudio, Boston, MA, USA) or GraphPad Prism6 (GraphPad Software, San Diego, CA, USA). For statistical comparisons, one‐way anova, two‐tailed paired Student's *t*‐test or unpaired Student's *t*‐test was used. *P* values of <0.05 were considered to be significant. Data are presented as mean ± standard deviation.

## Results

### Kras‐driven NSCLC models show heterogeneity in the histopathology of lesions

**Figure 1:**
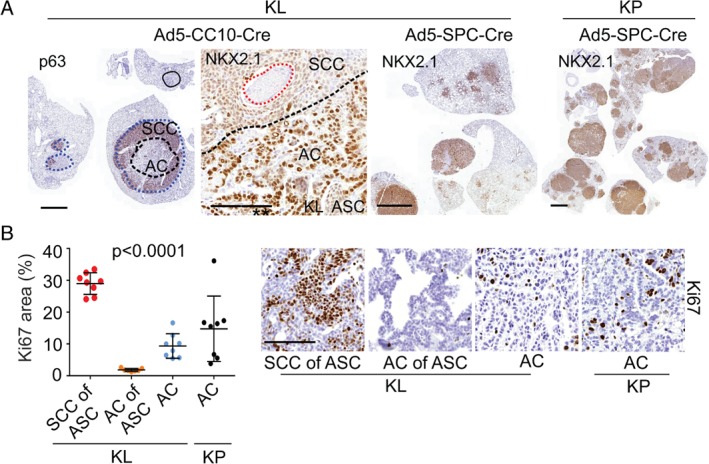
Histopathology‐related heterogeneity of KL and KP NSCLC tumours. (A) IHC images depict p63 and NKX2.1 in KL and KP tumours. The solid line indicates mucinous AC, the dotted lines (blue) indicate the ASCs and the AC cores of ASCs (black) or typical necrotic areas in ASC 33. Scale bars: 2 mm (low‐magnification images) and 100 μm (high‐magnification images). (B) Quantification and representative IHC images depicting Ki67 (stained area as percentage of whole tumour area) in KL and KP tumours (four to six analysed mice per tumour group; each dot represents one tumour). Scale bar: 100 μm. One‐way anova (average per mouse was used as an experimental unit). p < 0.0001. Data are shown as mean ± standard deviation.

We recently demonstrated that, in KL‐driven NSCLC, the spectra of induced histopathologies depend on the cell of origin 22. To study phenotypic variability and its dependence on genotype, in the current study we used both KL and KP models, which were infected with two progenitor cell type‐restricted adenoviruses. End‐stage lung tumours were analysed with immunohistochemistry and digital pathology tools. In line with our previous study 22, Ad5‐CC10‐Cre‐infected KL mice predominantly developed large ASC lesions with an inner core of NKX2.1^+/–^ (also known as TTF‐1) AC surrounded by a squamous p63^+^ region, as well as occasional p63^+/–^ NKX2.1^+^ mucinous or acinar ACs lesions, whereas Ad5‐SPC‐Cre‐infected KL mice predominantly developed papillary NKX2.1^+^ AC (Figure 1A). In KP mice, we observed the formation of p63^–^ NKX2.1^+^ ACs in a manner independent of the progenitor cell (Figure 1A), consistent with a previous report 19. Subsequently, we considered AC lesions of various histopathology subtypes as one group per genotype, and compared these two groups (KL AC and KP AC) with the ASCs formed in Ad5‐CC10‐Cre infected mice (KL ASC), subdividing the latter into their SCC and AC compartments.

We first assessed proliferation by quantifying IHC images for Ki67, and found that proliferation differed strongly across our four groups (anova *F*‐test, *p* < 0.0001; Figure 1B), with the SCC component of KL ASC lesions showing particularly high proliferation. We also noticed residual within‐group variation in proliferation, particularly in KP ACs (Figure 1B). Furthermore, ASC tumours often contained necrotic islets, which constitute a known clinical feature of ASC and SCC (Figure 1A). Thus, KP and KL models, despite being oncogenetically homogeneous, give rise to a spectrum of NSCLCs with varying histopathological and proliferation features.

### Histopathology‐specific variation in signalling pathway activities in NSCLCs

**Figure 2:**
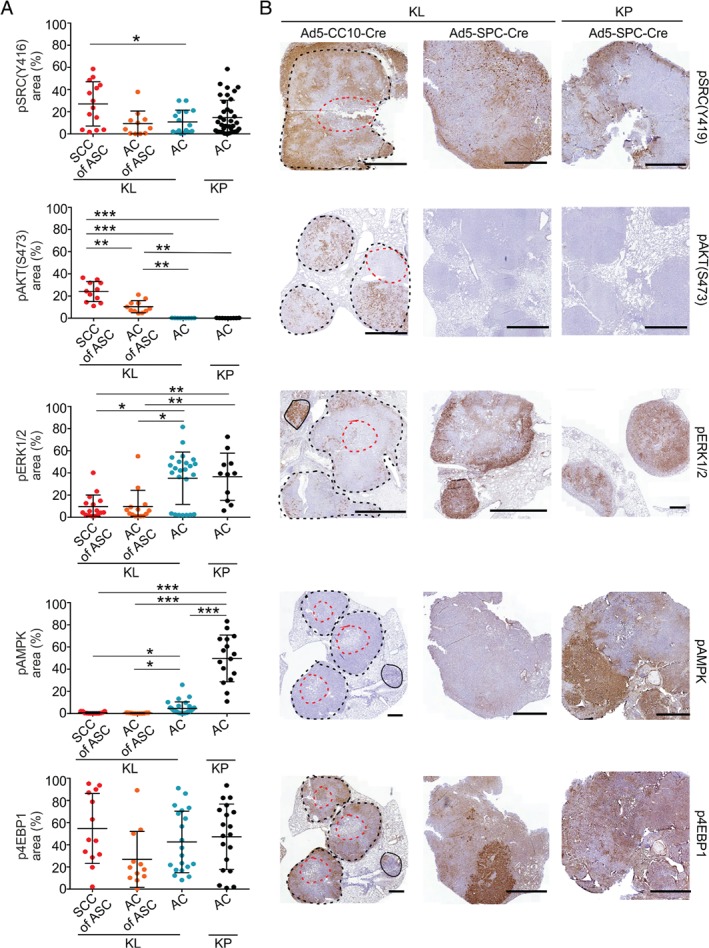
Oncogenic signalling pathway phosphoproteins show heterogeneous spatial distribution in KL and KP NSCLCs. (A) Quantified expression (percentage of tumour area stained) of pSRC(Y416), pAKT(S473), pERK1/2, pAMPK and p4EBP1 in Ad5‐CC10‐Cre‐induced KL SCC or AC of ASC tumour regions and Ad5‐SPC‐Cre‐induced KL or KP AC tumours. Each dot represents a tumour (11–29 tumours from seven mice for KL ASC, and five mice each for KL and KP AC), and lines and whiskers indicate mean ± standard deviation of the average values of individual tumours per histotype group. Significance was assessed with t‐tests (average per mouse was used as an experimental unit): *p < 0.05, **p < 0.01, ***p < 0.001. (B) IHC images of stained tumours induced by Ad5‐CC10‐Cre (KL) or Ad5‐SPC‐Cre (KL and KP); one image for each of the phosphoproteins and lesion groups is shown by a beeswarm plot in (A). For Ad5‐CC10‐Cre KL tumours, the solid line indicates mucinous AC, and black and red dotted lines define outer SSCs and inner AC cores of ASCs, respectively. Scale bars: 1 mm.

We next compared oncogenic signalling activities (supplementary material, Figure S1A) in the four groups (KP AC, KL AC, KL SCC of ASC, and KL AC of ASC) by staining for phosphorylated extracellular signal‐regulated kinase (pERK) 1/2 [mitogen‐activated protein kinase (MAPK) pathway], pAKT(S473) and p4EBP1 [phosphoinositide 3‐kinase (PI3K)–mammalian target of rapamycin (mTOR) pathway], and pSRC(Y416) and phosphorylated AMP‐activated protein kinase (pAMPK) [liver kinase B1 (LKB1)–mTOR pathway]. Marked differences were detected both between and within lesion groups (Figure 2A,B): the SCC regions of KL ASCs showed increased expression of pSRC as compared with ACs, and ASCs showed exclusive expression of pAKT; in contrast, pERK expression was stronger in AC‐histotype tumours. Consistent with LKB1‐dependent phosphorylation of the energy‐sensing kinase AMPK, pAMPK expression was decreased in KL tumours, as reported previously 24. Phosphorylation of the translational repressor 4EBP1, indicating activation of the mTOR complex 1 (mTORC1) biomass‐regulating pathway, showed high intertumour heterogeneity across groups.

Next, we investigated how representative signalling in murine NSCLCs is of clinical tumours. We therefore performed IHC analysis of a TMA encompassing human SCCs (28), ASCs (13) and papillary ACs (25) for pAKT and pERK (supplementary material, Figure S1B and Table S3). Tumour samples (*n* = 10) for which replicate punches from different subregions were included in the TMA showed variable pAKT or pERK expression, indicating spatial signalling heterogeneity in larger tumours. We also determined the LKB1 and TP53 status, following previously validated IHC analysis 13, 20, 22; whereas most tumours showed varying but positive nuclear TP53 protein accumulation, suggesting TP53 pathway mutation, LKB1 expression varied across NSCLC pathologies. Consistent with murine findings, absence of LKB1 expression was found in all three types of NSCLC pathology. In line with murine NSCLC, human AC tumours expressed pAKT rarely (one of 25 tumours), and significantly less often than ASC tumours (5/13; Fisher's test comparison with AC tumours, *p* = 0.012) and SCC tumours (8/28; Fisher's test comparison with AC tumours, *p* = 0.026). pERK expression was also examined; here, the majority of AC tumours (20/25) and ASC tumours (9/13) were positive for pERK, but significantly fewer SCC tumours were positive for pERK (13/28; Fisher's test comparison with AC tumours, *p* = 0.02) (supplementary material, Figure S1B and Table S3). Thus, both murine and human NSCLCs showed marked differences in signalling profiles, with an important part, but far from all, of this intertumour variability being explained by histotype: SRC, and even more so PI3K–AKT pathway activity, is more frequently detected in ASC histotype tumours, particularly in the SCC regions, whereas MAPK activity is more frequently detected in AC histotype tumours.

### Culture‐induced alterations in DNA damage, proliferation, and oncogenic signalling

Diagnostically informative disease models should reflect the between‐tumour and within‐tumour signalling heterogeneity. Before describing our drug perturbation experiments, we first discuss our tests performed to analyse to what extent cultured precision‐cut tumour slices maintain the proliferative and signalling features of the *in situ* tumour. This builds on our previous work 4, which showed (for KL ASC slices) that rotating incubation units achieve optimal tissue viability through intermittent medium immersion and oxygen exposure (supplementary material, Figure S2A).

In samples cultured for 72 h, we found that the medium‐exposed section of each slice, in particular, maintained viability (supplementary material, Figure S2B); KL AC slices often showed a culture‐induced reduction in viability (supplementary material, Figure S2C). Epithelial integrity was maintained, as shown by sustained E‐cadherin expression (supplementary material, Figure S2D). Next, we compared functional biomarkers in slices analysed at culture onset with those in directly neighbouring slices that had been cultured. After 24 h of culture, an increase in γH2AX‐marked DNA damage was visible (supplementary material, Figure S3A). Furthermore, whereas Ki67 was representative of native tissue in 0‐h slices (supplementary material, Figure S3B), Ki67 showed a gradual decrease in AC slices, whereas KL ASC slices revealed histotype‐specific alterations: SCC regions already showed decreased proliferation at 24 h, whereas AC regions showed increased epithelial cell proliferation starting 48 h after culture onset (supplementary material, Figure S3C). This suggests that the inner AC core of ASC tumours is particularly sensitive to slicing‐induced damage, perhaps akin to a wound‐healing response.

Next, we compared signalling activities in a panel of *in vivo* tumours (instantly fixed) to slices analysed at 0 h (fixed 90–120 min after mice had been killed). No significant alterations in pAKT, pERK, p4EBP1 or pSRC expression were detected in 0‐h slices (supplementary material, Figure S4A), or in slices cultured for 8 h (supplementary material, Figure S4B), indicating that pathway activities at culture onset or following short‐term culture represent the native tumours. However, alterations were measured in slices cultured for 24–48 h: selective pAKT induction was detected in a number of ASC samples at 24 h (supplementary material, Figure S5A), whereas this was never seen in ACs (supplementary material, Figure S6A), and pERK expression showed variable changes in both directions (supplementary material, Figure S5A). The latter is probably explained by different pERK expression in the compared neighbouring slices before culture, as pERK shows strong spatial heterogeneity at small scales (supplementary material, Figure S4C). The most prominent alterations, detected at 24 and 48 h in all lesions, were mTORC1 hyperactivation as measured by p4EBP1 induction, and SRC hyperactivation (supplementary material, Figures S5A and S6A). These alterations were observed regardless of medium supplementation with fetal bovine serum (used routinely) or autologous serum, or the use of serum‐free conditions (used for drug perturbations) (supplementary material, Figure S6B).

Finally, we investigated whether these dynamic signalling changes were peculiar to murine NSCLC slices. Assessment of PTEN loss‐driven murine prostate tumour slices similarly showed rapid pERK induction following slice culture (supplementary material, Figure S7A). Furthermore, slices from freshly resected clinical tumours showed elevated p4EBP1 and sustained or increased pERK expression in both NSCLC (supplementary material, Figure S5B) and prostate tumours (supplementary material, Figure S7B).

Overall, this shows that, whereas NSCLC slices maintain viability during 72 h of *ex vivo* culture, marked biological changes can be seen at the 24‐h time point. Therefore, tumour slices appear to constitute a good *ex vivo* model for *in situ* biology only for the first day. Then, however, the ability of slices to model the *in situ* spatial heterogeneity still makes them a valuable complement to cell lines, which are more stable but also unrealistically uniform.

### Combination therapy with MAPK and PI3K–mTOR inhibitors elicits cytotoxic responses

**Figure 3:**
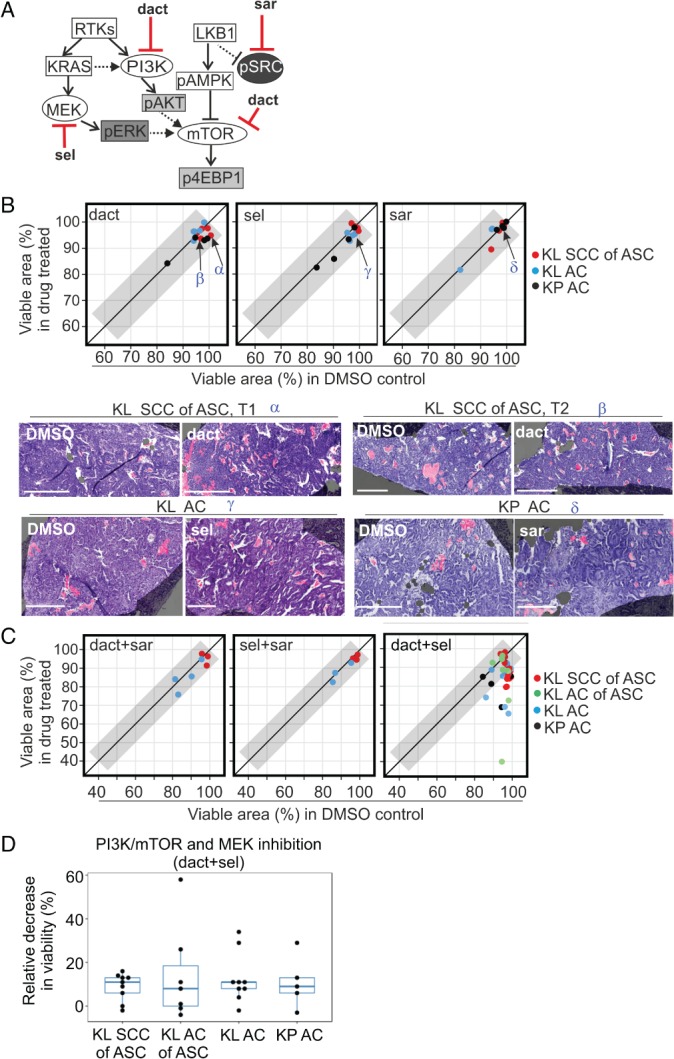
KL and KP tumour slices show cytotoxic responses to combined targeting of the MAPK and PI3K–mTOR pathways. (A) Signalling diagram depicting molecular compounds and their targets utilised in this study. (B) Responses following 24 h of treatment with single compounds: 0.5 or 1 μm dact, 0.5 μm sel, or 1 μm sar. The viable tissue area (percentage of tumour area) in drug‐treated slices is correlated with the viable area in neighbouring DMSO‐treated controls. The diagonal line corresponds to equal viability, and the grey area corresponds to the region with <5% difference between treated and control slices. Representative H&E images of matching DMSO‐treated and drug‐treated slices are shown; regions of cell death are pseudo‐masked in pink, and viable areas in purple. Scale bar: 300 μm. Greek letters (α, β, γ, δ) and arrows in the scatterplot indicate the tumours shown in the images. KL ASC samples represent data measured in larger SCC histotype regions; no visible response was detected in core KL ACs of ASC regions. (C) Correlation of viable tissue area in drug‐treated and matching DMSO control slices (percentage of total tumour area) in KL and KP tumours following 24 h of combination treatment with dact + sar (0.5 or 1 μm + 1 μm), sel + sar (0.5 μm + 1 μm), or dact + sel (0.5 or 1 μm + 0.5 μm). Of these, only the dact + sel combination elicited cytotoxicity, which was quantified for the four histotype groups. (D) Comparison of drug responses after 24 h of dact + sel treatment in the four different histotype groups: decrease in viability (%) measured as the ratio of the viable area in the drug‐treated slice and the matching DMSO control. One‐way anova was used for statistical comparison.

We now return to the central question of how the observed intertumour and intratumour heterogeneities in signalling affect pharmacodynamics. In light of the above findings, we restricted ourselves to immediate and short‐term treatments, again comparing adjacent slices, now treating one with a drug and the other with vehicle control. We chose three compounds with reported efficacy in preclinical studies on *Kras*‐driven NSCLC 14, 25, namely the dual PI3K and mTOR inhibitor NVP‐BEZ235 or dact, the MEK inhibitor AZD6244 or sel, and the SRC inhibitor sar (Figure 3A). The MAPK, PI3K–AKT and LKB1–AMPK pathways all regulate mTORC1 signalling to control cell growth and survival 26, 27, and loss of LKB1 activates FAK–SRC signalling to regulate invasiveness 14 (Figure 3A). First, close to minimally effective concentrations at which compounds suppressed their targeted pathways were identified, with suppressed p4EBP1 and pAKT (for dact), pERK (for sel) and pSRC (for sar) following 1–2 h or 24 h of treatment as read‐outs (supplementary material, Figure S8). Pathway inhibition was commonly already detected following 2 h of treatment, at which point signalling in DMSO‐treated control slices was still similar to that in uncultured slices (supplementary material, Figure S8B).

Next, pairs of neighbouring tumour slices were cultured either with compound or with DMSO, and the viable fractions (%) of the tumour area in the slices' top sections were quantified, in contrast to necrotic regions (supplementary material, Figure S9). We compared this with neighbouring uncultured (0 h) slices, in which relevant phosphoprotein markers were quantified (as percentage of tumour area). The raw data from all perturbation studies are shown in supplementary material, Table S4. We found that 24 h of treatment with single compounds did not elicit noticeable cytotoxic responses in any of the tumour groups (Figure 3B; difference from DMSO of <5% for all samples). Interestingly, only one of the three combinations, namely dact + sel, elicited any substantial response (Figure 3C; a difference from DMSO of >5% was found for four of nine ASC tumours, five of nine KL AC tumours, and two of six KP AC tumours). This was not accompanied by overt cytostatic responses, as no differences in Ki67 were measured between drug‐treated and DMSO‐treated controls (supplementary material, Figure S10A). Finally, the addition of sar to target SRC signalling enriched in ASCs did not enhance cytotoxicity, as responses to treatment with dact + sel + sar were similar to those to treatment with only dact + sel (supplementary material, Figure S10B). We conclude that NSCLC slices of both KL and KP genotypes, and regardless of AC or ASC pathology, show a lack of sensitivity to single pathway inhibition, but show selective sensitivity to combination treatment with MAPK and PI3K–mTOR inhibitors.

### Spatial aspects of oncogenic signalling correlate with response to combination therapy

**Figure 4:**
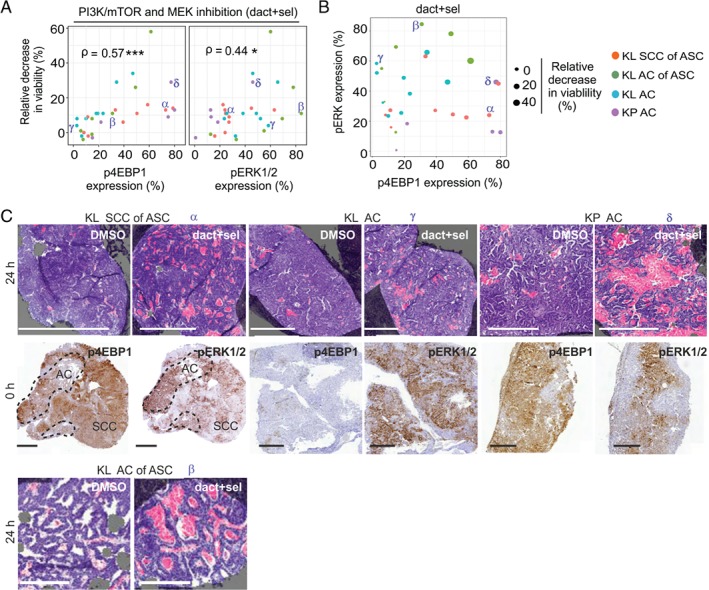
Oncogenic signalling activities at treatment onset correlate with dact + sel combination treatment responses. (A) Drug responses following 24 h of dact + sel treatment in slices representing KL SCC of ASC, KL AC of ASC, KL AC and KP AC tumour tissue, plotted against expression (percentage of tumour area) of p4EBP1 or pERK1/2, indicating targeted pathway activities in source tumours at treatment onset. Viability following drug treatment is depicted as relative decrease in viability (%). Spearman coefficients (ρ) are indicated with significance: *p < 0.05, ***p < 0.001. Greek letters (α, β, γ, δ) indicate tumour samples shown in (C). (B) Scatterplot depicting the correlation of pERK (y‐axis) and p4EBP1 (x‐axis) at treatment onset in relation to the relative decrease in viability (%; balloon size) in dact + sel‐treated slices after 24 h. The four tumour groups are indicated by colours; α, β, γ, δ indicate the tumour samples shown in (C). (C) Selected phosphoprotein expression and drug response data from slice experiments plotted in (A) and (B), indicated by Greek letters (α, β, γ, δ). Shown are representative images of H&E‐stained sections with masks for dead (pink) and viable (purple) tissue, and IHC stains of p4EBP1 and pERK in the corresponding 0‐h slices. Scale bars: 500 μm (top row) and 300 μm (bottom row).

We subsequently investigated whether responses following dact + sel treatment were determined by lesion‐specific targeted pathway activities. As no significant differences in viability reduction were detected between histotype groups (Figure 3D), these groups were pooled for the subsequent analyses, which correlated drug responses with MAPK and PI3K–mTOR activities; pAKT was excluded, as it was absent in most KL ACs of ASC regions and all pure ACs, implying that it does not contribute to drug sensitivity (supplementary material, Table S4). We compared the areas (%) of p4EBP1 and pERK expression in uncultured (0 h) slices, indicating *in situ* signalling pathway activities, with the response to 24 h of dact + sel treatment in neighbouring slices (supplementary material, Table S4). Increased p4EBP1 expression correlated most clearly with drug response (*p* < 0.001), but pERK expression also showed a significant correlation (*p* < 0.05) (Figure 4A). Dissection of targeted pathway activities showed concomitantly increased expression of both pERK and p4EBP1 specifically in samples with the most significant responses (>20% relative decrease in viability) (Figure 4B,C; α, β, δ), whereas more resistant samples showed selective activation of either pERK or p4EBP1 (Figure 4B,C; γ). Hence, our data suggest that increased activities of mTORC1 and MAPK sensitise to combined MAPK + PI3K–mTOR inhibition.

**Figure 5:**
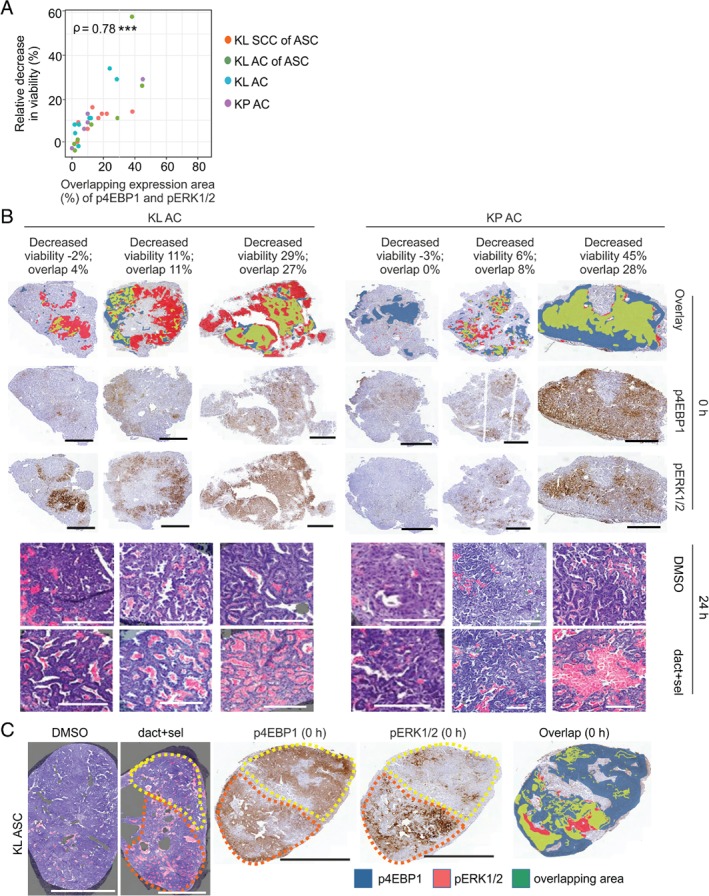
Combination treatment response is determined by spatial distribution of the targeted pathway activities in tumour slices. (A) Correlation of dact + sel treatment with the areas of p4EBP1 and pERK overlap (%) in neighbouring 0‐h slices in the four different tumour groups. Drug response depicted as relative decrease in viability (%) is measured as the ratio of quantified viable area in drug‐treated and corresponding DMSO‐treated controls. Spearman coefficients (ρ) are indicated with significance: *p < 0.05. (B) IHC images depicting p4EBP1 expression (blue), pERK expression (red) or their overlapping expression (green) at 0 h in three KP or KL AC tumours selected from the data plotted in (A) and (B). H&E images of drug‐treated and matching DMSO‐treated controls are pseudo‐marked to indicate dead (pink) and viable (purple) areas. Scale bars: 1 mm (immunohistochemistry) and 200 μm (H&E). (C) H&E images of DMSO‐treated or dact + sel‐treated slices showing dead (pink) and viable (purple) areas of a KL SCC of an ASC tumour selected from the data plotted in (A) and (B). IHC images depicting p4EBP expression (blue), pERK expression (red) or their overlapping expression (green) at treatment onset. The yellow dotted line outlines an area with low cytotoxicity (H&E image), lower pERK expression (IHC image) and lower phosphoprotein overlap than in the area marked by the orange dotted line. Scale bars: 500 μm.

Finally, we wished to more conclusively address whether responses were related to increased sensitivity of individual cells dually active for the targeted pathways, and investigated how the spatial distribution of p4EBP1 and pERK expression determined drug responses. We therefore quantified the area of overlap between these phosphoproteins at treatment onset, and correlated this with drug‐induced cytotoxicity. This revealed a significant correlation (*p* < 0.001) between the spatial overlap (percentage total tumour area) of pERK and p4EBP1 expression and dact + sel responses (Figure 5A,B); samples in which the extent of signalling overlap was smaller (<15%) were more resistant. Interestingly, whereas the majority of AC samples with increased signalling overlap (>15%; six samples) showed significant drug responses, KL SCCs of ASC samples with similar signalling overlap showed lower drug responses (>15%; four samples), tentatively suggesting an SCC histotype‐specific resistance mechanism (Figure 5A). Consistent with the thesis that a spatial correlation exists between combination drug response and signalling, analysis of individual ASC samples showed intratumour regional differences in responses: regions with larger areas of mTORC1 and MAPK signalling overlap showed increased cytotoxicity (Figure 5C), and the AC cores of ASCs showed increased cytotoxicity as compared with the outer SCC regions in the same tumour (supplementary material, Figure S10C). In conclusion, we observed pronounced intralesional spatial heterogeneity in *Kras*‐associated signalling activities, resulting in sensitivity to combined inhibition of PI3K–mTOR and MAPK pathways, particularly in samples and tumour subregions in which both targeted pathways are active.

## Discussion

It is increasingly recognised that both phenotypic and genetic diversities govern tumour evolution 2. Although pharmacological screens are feasible on patient‐derived cultures 28, these, as well as more routinely applied diagnostic assays, disregard such spatial heterogeneities. We present here our analysis of mutant *Kras*‐related signalling in NSCLCs. Unexpectedly, we found that, whereas genetic drivers define histopathology spectra, most of the signalling variance in both murine and human tumours is explained by histopathology. Specifically, increased PI3K–AKT and SRC activities were more frequently detected in ASCs, whereas MAPK activity was more selective for ACs. In addition, pronounced intertumour and intratumour heterogeneity in MAPK and biomass‐regulating mTORC1 activities were observed, possibly being explained by intratumour variation in mutant *Kras* copy gains, which are known to affect MAPK activity and glucose metabolism 29, 30, or regional differences in nutrient availabilities. Importantly, the finding that signalling variation in NSCLC relates to histopathology matches our identification of histotype‐specific immune microenvironments in KL‐driven NSCLC 22, and, together with reported differences in energetic dependencies 31, 32 and epigenetic signatures 33, warrants consideration of histotype‐specific phenotypes in treatment decisions.

The establishment of a tumour's histopathology is the culmination of a complex interplay between genetic drivers, the tumour cell of origin, somatic gene alterations, and factors in the microenvironment. Therefore, the finding that signalling appears to be specific to NSCLC histotypes across species is tantalizing. Although analysis of a larger set of samples is required, this indicates a causative role for signalling in the formation of histopathology subtypes. We detected both restrictive PI3K–AKT activity and increased SRC activity in ASC lesions. This extends the finding of prior studies that ascribed altered PI3K–AKT and SRC signalling to *Lkb1* loss in *Kras*‐driven NSCLC 14, 15, but we relate this to an alteration in histotype spectra, particularly an increase in ASCs. Interestingly, mutations in the PI3K pathway have been detected specifically in the SCC, but not in the AC, components of clinical ASCs 34. This implies that PI3K–AKT signalling could indeed drive human squamous tumours, and corroborates findings demonstrating SCC induction following loss of the PTEN negative regulator of PI3K–AKT signalling 35, 36.

We used a slice explant model optimised for pulmonary, breast and prostate cancer tissues 4 to investigate how signalling heterogeneity in individual lesions influences drug responses. NSCLC slices cultured on rotating incubation units, to ensure continuous oxygenation, were fragile and showed dynamic culture‐induced changes, most prominently altered proliferation and mTORC1 hyperactivation. Although previous reports showed sustained viability of clinical tumour slices for 4–14 days 6, 7, 9, 10, these studies derived conclusions from the selective analysis of cultured slices, whereas we systematically compared neighbouring cultured and uncultured slices. Furthermore, our study is the first to deeply analyse intratumour oncogenic signalling, and shows that signalling and proliferation alterations take place prior to overt changes in viability. Culture in serum‐free medium improved neither viability nor signalling changes. Notably, increased activity of mTORC1, an inducer of protein synthesis, was also detected in clinical tumour slices. Thus, metabolic adaptation appears to be common to culture of primary tissue, which corroborates reports showing that nutrient dependencies *ex vivo* differ from those of native NSCLC tumours 30, 37.

We provide evidence that the ability of slices to, at least temporarily, model *in situ* spatial heterogeneity makes them of unique diagnostic value. Specifically, responses to combined MAPK and PI3K–mTOR inhibitor treatment closely correlated with the spatial activities of both targeted pathways. Further affirming the value of slices, our results on *ex vivo* inhibition of KRAS effector pathways align with published preclinical data; the finding that AC slices show selective sensitivity to dact + sel combination treatment agrees with the observation that combined MAPK + PI3K–mTOR inhibition reduces the AC tumour burden of *Kras* ^*G12D*^ mice more effectively than single pathway inhibition 25. Furthermore, KL mice with mixed ASC and SCC spectrum tumours were shown to be largely unresponsive to dact + sel treatment 14. This is consistent with low MAPK activity in ASC tumours, and resistance of, particularly, the larger SCC regions to combination treatment in slice explants. Interestingly, despite activation of SRC signalling in ASC tumours, inclusion of sar did not exacerbate cytotoxicity in ASC slices, which deviates from the regression of KL‐driven tumours following dact + sel treatment in combination with the non‐selective SRC inhibitor dasatinib 14. This discrepancy is probably explained by the role of SRC activity in invasion 38, and possibly also a role of antitumour T‐cell responses in dasatinib's efficacy 39, as these demand *in vivo* modelling. Together, our data support the further development of slices to assess lesion‐specific oncogenic pathway dependencies, and suggest that their use may complement preclinical studies on heterogeneous solid tumours.

Although all studied GEMM tumours are driven by oncogenic *Kras*, strikingly, signalling activities, including that of MAPK, are highly variable from tumour to tumour. Taken together with, first, our finding that overlapping signalling activities dictate combination response, and second, the detection of similar signalling variabilities in clinical NSCLCs, this suggests that spatially defined phenotypic heterogeneity may also influence treatment efficacy in clinical samples. Deeper dissection of signalling heterogeneity in the context of clinical NSCLC is particularly relevant for KL‐driven tumours: no targeted inhibitors are known for this disease subtype, and LKB1‐mutant NSCLCs have been shown to have an ‘immune‐inert’ state of low programmed death‐ligand 1 (PD‐L1) checkpoint protein 13, 39, 40, making them unsuitable for immunotherapy. Therefore, future efforts should investigate how short‐term slice cultures derived from patient tumours can be used to study the impact of tumour‐intrinsic phenotypic variation on drug responses to clinically prescribed therapies, or to compounds inhibiting novel targets, or to validate the efficacy of combination strategies. Overall, our study suggests that analysis of the spatial activities of oncogenic functions, such as signalling activities, provides important diagnostic value and should be used in addition to routinely applied molecular and immunotherapy biomarkers.

In conclusion, we show that both the cell of origin and genetic drivers play important roles in establishing heterogeneous activities of commonly targeted oncogenic signalling pathways, implying the existence of NSCLC histopathology‐specific signalling networks. Our findings caution against an over‐reliance on genetic biomarkers in diagnostic settings, particularly if mutation‐targeted therapies are lacking. Furthermore, our study underscores a need for, and informs on, diagnostic assays to assess spatial signalling heterogeneity when making personalised therapeutic decisions.

## Author contributions

KN, WS, and EWV planned the experiments. KN, SA, and EWV wrote the manuscript. KN, ASN, EP, PvD, VU, and AH performed the experiments. TaH planned the experiment on human prostate tissue. MIM and KS performed pathological review. RT quantified necrosis masks. JLa, TaH, JR, JLu, and MIM provided technical and material support. KN, ASN, KS, and SA analysed the data. OK, AR, JT, NL, and EWV provided supervision.

**SUPPLEMENTARY MATERIAL ONLINE**

**Supplementary materials and methods**

**Supplementary figure legends**

**Figure S1.** Intratumoral signaling heterogeneity in NSCLC tumors

**Figure S2.** Workflow for tumor slicing, culture and analysis

**Figure S3.** DNA damage induction and altered cell proliferation in cultured KL and KP slices

**Figure S4.** Oncogenic signaling activities *in vivo* and in freshly cut and short‐term cultivated tumor slices

**Figure S5.** Dynamic alterations in *ex vivo* oncogenic signaling pathway activities

**Figure S6.** Altered oncogenic signaling activities in cultivated KL and KP slices

**Figure S7.** Altered oncogenic signaling in prostate tumor slices

**Figure S8.** Definition of minimally effective drug concentrations able to inhibit oncogenic signaling in tumor slices

**Figure S9.** Cytotoxic effects of short‐term targeted therapy in ASC and AC slices

**Figure S10.** Dact+sel combination treatment induces cytotoxicity, and this is not enhanced by sar addition

**Table S1.** Slice culture conditions and methods for different tumor pathologies

**Table S2.** Antibody details and verification

**Table S3.** Human NSCLC TMA analyses

**Table S4.** Raw data related to Figure 4 and Figure 5

## Supporting information


**Supplementary materials and methods**

**Supplementary figure legends**

**Figure S1.** Intratumoral signaling heterogeneity in NSCLC tumors. (A) Effector pathways downstream of KRAS and LKB1. Boxes filled in grey or black indicate phosphoproteins used in the study for pathway activity readouts. Dotted and solid lines indicate indirect and direct regulation, respectively. (B) Representative IHC images depicting pERK, pAKT, LKB1, and p53 in human lung tumor TMA encompassing AC, ASC, and SCC samples. Absence of LKB1 expression and nuclear p53 accumulation possibly indicates genetic alteration in LKB1 gene and p53 pathway respectively. (++) = strong and uniform expression, (+): = weak or mosaic expression, (‐) = absence of expression. Scale bar: 1 mm.

**Figure S2.** Workflow for tumor slicing, culture and analysis. (A) Schematic depicting the precision cutting of 200 μm slices, cultivation using a rotating incubation unit, and analysis of the top most medium‐exposed sections of individual slices analyzed with digital pathology tools. The incubation unit permits intermittent immersion of slices into media, required for optimal viability of murine NSCLC slices cultivated for 24 h 3. (B) Representative images of H&E‐stained sections with masks for dead (pink) and viable (purple) tissue showing formation of culture‐induced necrosis in KL ASC slices during 72 h culture. The top layer of cultivated slices (marked by the orange box) resembles best the 0 h baseline slices and was thus selected for quantitative analyses. Scale bars: 1 mm. (C) Quantitation of the culture‐induced reduction in viable tissue area. Shown is the ratio of viable tissue area in cultivated slices (% of total area; measured in H&E stained sections of the slices' top layers) to the viable tissue area (% of total area) in neighboring uncultured (0 h) slices; bars show mean ± SD of these ratios, from 6‐7 sliced tumors per tumor group. At the 24 h time point, KL ACs show often decreased viability compared with the KL ASC and KP AC tumors (Student's t‐test, p < 0.05). (D) Representative IHC images depicting E‐cadherin expression in KL ASC, KL AC or KP AC slices at the indicated time points, representing the top layer of cultivated slices. Results are representative of three independent experiments per group. Scale bar: 100 μm.

**Figure S3.** DNA damage induction and altered cell proliferation in cultured KL and KP slices. (A) Nuclear γH2AX (% of tumor area) in KL and KP tumor slices, comparing neighboring 0 h (black circle) and cultured (red square) slices. Each linked pair represents one individual tumor, and tumors were collected from 3‐5 mice for each histotype group. A two‐tailed t‐test was used for pairwise statistical comparison for each group's culture time, and if slice number > 2; * p < 0.05, ** p < 0.01, *** p < 0.001. (B) Statistical comparison of Ki67 (% area) in KL and KP in vivo tumors (black) and freshly cut (0 h) slices (red). Each data point represents one tumor and samples were collected from 4‐6 mice per group (tumors) or 3‐5 mice per group (slices). The data demonstrate that, compared to in vivo tumors, Ki67 expression is not altered in 0 h slices. Two‐tailed (paired) t‐test was used for statistics and data are shown as mean ± SD. (C) Nuclear Ki67 (% of tumor area) in KL and KP tumor slices. Each linked pair represents an individual tumor, and tumors were collected from 3‐5 mice per group. Two‐tailed t‐test was performed as pairwise comparison if slice number > 2 per time point; * p < 0.05, ** p < 0.01, *** p < 0.001. Images depict representative Ki67 staining in the SCC and AC components of baseline 0 h and cultured KL ASC slices from one tumor. Scale bar: 50 μm.

**Figure S4.** Oncogenic signaling activities in vivo and in freshly cut and short‐term cultivated tumor slices. (A) Statistical comparison of pAKT, pERK, p4EBP1, and pSRC, (% area) in separate panels of KL and KP in vivo tumors (black) and freshly cut (0 h) slices (red). Each data point represents one tumor and lesions were harvested from 5‐7 mice per tumor group for each phosphoprotein. Two‐tailed (unpaired) t‐test was used for statistics and data are shown as mean ± SD. (B) IHC for pSRC(Y416), p4EBP1, pERK1/2, and pAKT(S473) at 0 h, 8 h, and 24 h following culture onset in KL ASC slices. Images are representative of three independent experiments. Scale bar: 1 mm. (C) Intra‐tumor heterogeneity of p4EBP1 and pERK1/2 expression quantitated in different tumor layers (A, B, and/or C) within three KL and KP AC tumors (t1‐t3). IHC staining of pERK1/2 expression in one KP AC tumor reveal higher expression area in the B layer than in the A layer which is closer to the tumor surface.

**Figure S5.** Dynamic alterations in ex vivo oncogenic signaling pathway activities. (A) Analysis of pERK1/2, p4EBP1, and pSRC(Y416) (% area) in KL ASC or AC and KP AC slices, as well as pAKT(S473) in KL ASC slices, comparing neighboring 0 h (black circle) and cultured (red square) slices. Each linked pair represents individual tumors, and tumors were collected from 3‐5 mice for each histotype group. A two‐tailed t‐test was used for pairwise statistical comparison for each group's culture time point; * p < 0.05, ** p < 0.01, *** p < 0.001. (B) Representative IHC images showing pERK1/2 and p4EBP1 in 0 h and 24 h culture time points of human AC and SCC histopathology NSCLC slices. Data plots depict quantitated epithelial biomarker expression during culture in three linked slice pairs representing the 0 h slice (circle) and cultured slice (square) cut from three NSCLC tumors (T1‐T3). Data are representative of 3‐4 technical replicates per tumor. Two of three cultured tumors revealed increased p4EBP1 and pERK. Asterisks indicate stroma, arrows and arrowhead point to pERK positive and pERK negative areas in the AC epithelium, respectively. The dotted line defines the SCC epithelium which in pERK and p4EBP1 stains (bottom row). Scale bar: 500 μm.

**Figure S6.** Altered oncogenic signaling activities in cultivated KL and KP slices. (A) Representative IHC images depicting pAKT (S473), pERK1/2, pSRC (Y416), or p4EBP1 in 0 h and 24 h culture time point slices derived from KL ASC, KL AC or KP AC tumors. n.a. = not available. Scale bars: 100 μm. (B) Representative IHC images depicting pAKT (S473), pERK1/2, pSRC (Y416), or p4EBP1 in 0 h and 24 h culture time point KL ASC slices cultivated with 10% fetal bovine serum (FBS), 10% mouse (autologous) serum (MS) or without serum. Absence of serum does not affect phosphoprotein expression. For serum level comparison and phosphoprotein expression, two tumors were sliced, cultivated and analyzed visually. Scale bar: 200 μm.

**Figure S7.** Altered oncogenic signaling in prostate tumor slices. (A) pERK1/2 and pAKT(S473) expression, following IHC or immunoblotting analysis, in murine Pten loss‐driven in vivo prostate tumors, freshly cut (0 h) and 24 h or 96 h cultured slices in the presence or absence of pAKT inhibitor (1 μM). Images are representative of two independent experiments. Scale bar: 50 μm. (B) Representative IHC analysis depicting p4EBP1 or pERK1/2 in human prostate tumor slices at 0 h or 24 h following culture onset. Data plots depict quantitated p4EBP1 and pERK1/2 (% area) in three sliced human prostate tumors (T1‐T3) at 0 h or following 24 h slice culture. Scale bar: 500 μm. Two‐tailed (paired) t‐test was used for statistical comparison (p < *0.05).

**Figure S8.** Definition of minimally effective drug concentrations able to inhibit oncogenic signaling in tumor slices. (A) IHC of p4EBP1 in a freshly cut (0 h) KP AC slice, or after PI3K/mTOR inhibition by treatment with 0.1 μM or 1 μM dact, for 1 h or 24 h. 1 μM, but not 0.1 μM, dact treatment effectively suppressed 4EBP1 phosphorylation at 24 h. Images are representative of two independent experiments. Scale bar: 1 mm. (B) IHC analysis of p4EBP1 and pAKT(S473) after 2 h treatment of KL ASC slices with 1 μM dact, or pERK1/2 after 2 h treatment of KL ASC slices with 0.5 μM sel, and comparative phosphoprotein expression in 0 h, 2 h, or 24 h DMSO‐treated slices. Both compounds effectively inhibited their targeted pathways following 2 h of treatment. Images are representative of two independent experiments. Scale bar: 1 mm. (C) IHC depicting pAKT (S473), pSRC(Y416) in KL ASC or pERK1/2 in KL AC slices, following 24 h treatment with DMSO or 1 μM dact, 1 μM sar, or 0.5 μM sel. 20‐40 tumors were analyzed per treatment experiment. All compounds effectively suppressed targeted signaling pathways following 24 h of treatment. While 1 μM dact treatment typically resulted in the partial inhibition of AKT phosphorylation (arrow points at residual signal), this fully inhibited 4EBP1 phosphorylation (shown in B). Scale bars: 1 mm. (D) IHC showing pSRC following 24 h treatment with 0.3 μM or 1 μM sar. 1 μM, but not 0.3 μM sar treatment effectively suppressed pSRC expression. Images are representative of four independent experiments. Scale bars: 500 μm.

**Figure S9.** Cytotoxic effects of short‐term targeted therapy in ASC and AC slices. H&E images of matching DMSO‐treated and drug‐treated slices of ASC and AC showing necrotic cells and apoptotic bodies in treated slices (arrows). Scale bar: 50 μm.

**Figure S10.** Dact+sel combination treatment induces cytotoxicity, and this is not enhanced by sar addition. (A) Quantitation of Ki67 (%) in DMSO control (black) and neighboring drug‐treated tumor slices (dact+sel in red; dact in blue; sel in grey). Representative IHC images depicting Ki67 in dact+sel‐treated and matching DMSO control KL ASC or KP AC slices. Scale bar: 100 μm. (B) Cytotoxic responses shown as relative decrease in viability (%) following 24 h treatment of KL SCC of ASC, KL AC, and KP tumor slices with dact+sel or sar + dact+sel. Drug responses are measured as the ratio of quantitated viable tissue area in drug‐ and matching DMSO‐treated slices. (C) Comparison of drug responses in the AC and SCC regions of KL ASC tissue slice samples following 24 h treatment with dact+sel. Drug responses are depicted as the relative decrease in viability (%), measured as the ratio of the quantitated viable tissue area in drug‐ and matching DMSO‐treated slices. The tumor sample indicated by an arrow shows increased drug response in the AC region compared with the SCC region; H&E images depict masked dead (pink) and viable (purple) areas. The yellow solid line (H&E) and dotted lines (IHC) outline the SCC area that shows decreased drug response and lower pERK compared with the AC area marked by red lines. Scale bars: 500 μm (H&E), 1 mm (IHC).

**Table S1.** Slice culture conditions and methods for different tumor pathologies

**Table S2.** Antibody details and verification

**Table S3.** Human NSCLC TMA analyses

**Table S4.** Raw data related to Figure 4 and Figure 5
