# Supplementary material for: Spatial aspects of oncogenic signalling determine the response to combination therapy in slice explants from Kras‐driven lung tumours
Source: J Pathol. 2018 Apr 2;245(1):101–13. doi: 10.1002/path.5059 (PMC5947161; doi:10.1002/path.5059)
Supplement: Supplementary file 1 — Supplementary materials and methods [file PATH-245-101-s001.docx]

**Supplementary materials and methods**

Citation numbers refer to the main text document

**Mice**

For tissue slice culture experiments using tumors generated in a prostate cancer GEMM, PSA-Cre mice (strain FVB) and mice carrying the *Pten-loxP* allele (strain 129Ola) were crossed to generate *PSA-Cre;Pten-loxP/loxP* (*Pten*) mice, as described previously [41]. Animal experiments performed with the *Pten* mice were approved by the animal experimentation committee of the Erasmus Medical Center (DEC-consult, permit number 106-05-11). *Pten* mice develop homogenous hyperplastic prostates around 4-5 months of age. Tumors progress to intraductal carcinoma and carcinosarcoma pathology at older age (> 10 months) [42], and these were selected for slice culture (see below).

**Blood serum preparation**

Blood was collected from terminally anesthetized moribund tumor-bearing KL mice (Mebunat, 100 mg/kg, i.p. injection, Orion Pharma, Espoo, Finland) via saphenous vein bleed or cardiac puncture, and serum was prepared by clotting blood for 1 h at room temperature and removal of the clot by centrifugation (1500 g/15 min, at 4 °C). For tumor slice culture, 10% serum was added to the culture medium.

**Human prostate cancer**

Prostate tissue was collected from patients undergoing resection of the malignant prostate at the Helsinki University Hospital, Finland. Samples were obtained from patients participating with informed consent in the Urological Biobank Initiative (Helsinki Urological Biobank; HUB) (Dnro 263/13/03/02/2011; 379/13/03/02/2012 and Dnro § 212). Freshly resected tumor pieces were processed for slicing and culture, followed by histological analysis at set time points.

**Murine and human tumor slice culture**

All human and mouse lung and prostate tumors were precision cut with vibrating blade microtome (Leica VT1200S). Prior to slicing, a Vibrocheck was performed to permit better sample quality. Dissected murine lung lobes were transported in cold salt solution (HBS) supplemented with glucose (30 mM Hepes/4 mM glucose/3 mM KCl/130 mM NaCl/1 mM Na_2_HPO_4_/0.0033 mM Phenol red Na salt). Murine lung tumors of 2-10 mm in diameter were selected for slicing by cutting the individual tumors from the lobes. Tumors were adhered to the magnetic specimen holder of a Leica VT1200S vibrating blade microtome, using cyanoacrylate adhesive. We found that clinical samples with a high proportion of normal tissue affected the ability to cut sufficient numbers of high quality slices, due to differences between tumor and normal tissue stiffness. The slicing speed and amplitude was adjusted for individual samples, due to variations in sample stiffness; for all murine slice experiments, 0.12 mm/s speed and 2.5-2.7 amplitude was used. Freshly cut tumor slices were collected in 24-well plates with cold HBS, while keeping track of the order in which slices were cut. Murine lung tumor slices were transferred to rotating culture incubators, within 90–120 min post sacrificing of the mouse. Resected patient lung tumor samples were selected by a pathologist, and arrived for slicing within three hours post-surgery; slices were placed in culture within the next 60–90 min. NSCLC tumor slices were cultured on rotating incubation units (Alabama Research & Development), as described in [43]. Human prostate tumor samples were cultured as described in [44]. Murine *Pten* prostate tumor slices were cultivated with or without an AKT inhibitor 1 μM GDC0941 (Pictilisib, Selleckchem). For all tumor slices, cultivation was performed at 37 °C and 5% CO_2_, in a humidified incubator under atmospheric (21%) oxygen. More details on the culture conditions for the different pathologies are given in supplementary materials, Table S1. Slices were harvested at different time points, snap-frozen for protein isolation and immunoblotting (*Pten* tumor slices), or fixed in 4% formaldehyde overnight at 4 °C, followed by paraffin embedding for H&E staining or IHC.

**Immunohistochemistry**

The specificity of primary rabbit antibodies was validated according to the procedures depicted in Table S2.

**Immunoblotting**

Immunoblotting was performed according to standard procedures using 10 μg protein from *Pten* prostate tumor lysates. Primary antibody (anti-p-ERK1/2 and anti-p-AKT (S473), see supplementary materials, Table S2) incubation was performed overnight at 4 °C. Signals were visualized with Pierce Western Blotting Substrate (T) using polyclonal goat anti-rabbit immunoglobulins/horseradish peroxidase (Dako, Agilent Technologies, Glostrup, Denmark).
