# Supplementary material for: Spatial aspects of oncogenic signalling determine the response to combination therapy in slice explants from Kras‐driven lung tumours
Source: J Pathol. 2018 Apr 2;245(1):101–13. doi: 10.1002/path.5059 (PMC5947161; doi:10.1002/path.5059)
Supplement: Supplementary file 2 — Supplementary figure legends [file PATH-245-101-s002.docx]

**Coates edited**

**SUPPLEMENTARY FIGURE LEGENDS**

**Figure S1.** Intratumoral signaling heterogeneity in NSCLC tumors. (A) Effector pathways downstream of KRAS and LKB1. Boxes filled in grey or black indicate phosphoproteins used in the study for pathway activity readouts. Dotted and solid lines indicate indirect and direct regulation, respectively. (B) Representative IHC images depicting pERK, pAKT, LKB1, and p53 in human lung tumor TMA encompassing AC, ASC, and SCC samples. Absence of LKB1 expression and nuclear p53 accumulation possibly indicates genetic alteration in *LKB1* gene and p53 pathway respectively. (++) = strong and uniform expression, (+): = weak or mosaic expression, (-) = absence of expression. Scale bar: 1 mm.

**Figure S2.** Workflow for tumor slicing, culture and analysis. (A) Schematic depicting the precision cutting of 200 μm slices, cultivation using a rotating incubation unit, and analysis of the top most medium-exposed sections of individual slices analyzed with digital pathology tools. The incubation unit permits intermittent immersion of slices into media, required for optimal viability of murine NSCLC slices cultivated for 24 h [3]. (B) Representative images of H&E-stained sections with masks for dead (pink) and viable (purple) tissue showing formation of culture-induced necrosis in KL ASC slices during 72 h culture. The top layer of cultivated slices (marked by the orange box) resembles best the 0 h baseline slices and was thus selected for quantitative analyses. Scale bars: 1 mm. (C) Quantitation of the culture-induced reduction in viable tissue area. Shown is the ratio of viable tissue area in cultivated slices (% of total area; measured in H&E stained sections of the slices’ top layers) to the viable tissue area (% of total area) in neighboring uncultured (0 h) slices; bars show mean ± SD of these ratios, from 6-7 sliced tumors per tumor group. At the 24 h time point, KL ACs show often decreased viability compared with the KL ASC and KP AC tumors (Student’s t-test, p < 0.05). (D) Representative IHC images depicting E-cadherin expression in KL ASC, KL AC or KP AC slices at the indicated time points, representing the top layer of cultivated slices. Results are representative of three independent experiments per group. Scale bar: 100 μm.

**Figure S3.** DNA damage induction and altered cell proliferation in cultured KL and KP slices. (A) Nuclear γH2AX (% of tumor area) in KL and KP tumor slices, comparing neighboring 0 h (black circle) and cultured (red square) slices. Each linked pair represents one individual tumor, and tumors were collected from 3-5 mice for each histotype group. A two-tailed t-test was used for pairwise statistical comparison for each group’s culture time, and if slice number > 2; * p < 0.05, ** p < 0.01, *** p < 0.001. (B) Statistical comparison of Ki67 (% area) in KL and KP *in vivo* tumors (black) and freshly cut (0 h) slices (red). Each data point represents one tumor and samples were collected from 4-6 mice per group (tumors) or 3-5 mice per group (slices). The data demonstrate that, compared to *in vivo* tumors, Ki67 expression is not altered in 0 h slices. Two-tailed (paired) t-test was used for statistics and data are shown as mean ± SD. (C) Nuclear Ki67 (% of tumor area) in KL and KP tumor slices. Each linked pair represents an individual tumor, and tumors were collected from 3-5 mice per group. Two-tailed t-test was performed as pairwise comparison if slice number > 2 per time point; * p < 0.05, ** p < 0.01, *** p < 0.001. Images depict representative Ki67 staining in the SCC and AC components of baseline 0h and cultured KL ASC slices from one tumor. Scale bar: 50 μm.

**Figure S4.** Oncogenic signaling activities *in vivo* and in freshly cut and short-term cultivated tumor slices. (A) Statistical comparison of pAKT, pERK, p4EBP1, and pSRC, (% area) in separate panels of KL and KP *in vivo* tumors (black) and freshly cut (0 h) slices (red). Each data point represents one tumor and lesions were harvested from 5-7 mice per tumor group for each phosphoprotein. Two-tailed (unpaired) t-test was used for statistics and data are shown as mean ± SD. (B) IHC for pSRC(Y416), p4EBP1, pERK1/2, and pAKT(S473) at 0 h, 8 h, and 24 h following culture onset in KL ASC slices. Images are representative of three independent experiments. Scale bar: 1 mm. (C) Intra-tumor heterogeneity of p4EBP1 and pERK1/2 expression quantitated in different tumor layers (A, B, and/or C) within three KL and KP AC tumors (t1-t3). IHC staining of pERK1/2 expression in one KP AC tumor reveal higher expression area in the B layer than in the A layer which is closer to the tumor surface.

**Figure S5.** Dynamic alterations in *ex vivo* oncogenic signaling pathway activities. (A) Analysis of pERK1/2, p4EBP1, and pSRC(Y416) (% area) in KL ASC or AC and KP AC slices, as well as pAKT(S473) in KL ASC slices, comparing neighboring 0 h (black circle) and cultured (red square) slices. Each linked pair represents individual tumors, and tumors were collected from 3-5 mice for each histotype group. A two-tailed t-test was used for pairwise statistical comparison for each group’s culture time point; * p < 0.05, ** p < 0.01, *** p < 0.001. (B) Representative IHC images showing pERK1/2 and p4EBP1 in 0 h and 24 h culture time points of human AC and SCC histopathology NSCLC slices. Data plots depict quantitated epithelial biomarker expression during culture in three linked slice pairs representing the 0h slice (circle) and cultured slice (square) cut from three NSCLC tumors (T1-T3). Data are representative of 3-4 technical replicates per tumor. Two of three cultured tumors revealed increased p4EBP1 and pERK. Asterisks indicate stroma, arrows and arrowhead point to pERK positive and pERK negative areas in the AC epithelium, respectively. The dotted line defines the SCC epithelium which in pERK and p4EBP1 stains (bottom row). Scale bar: 500 μm.

**Figure S6.** Altered oncogenic signaling activities in cultivated KL and KP slices. (A) Representative IHC images depicting pAKT (S473), pERK1/2, pSRC (Y416), or p4EBP1 in 0 h and 24 h culture time point slices derived from KL ASC, KL AC or KP AC tumors. n.a. = not available. Scale bars: 100 μm. (B) Representative IHC images depicting pAKT (S473), pERK1/2, pSRC (Y416), or p4EBP1 in 0 h and 24 h culture time point KL ASC slices cultivated with 10% fetal bovine serum (FBS), 10% mouse (autologous) serum (MS) or without serum. Absence of serum does not affect phosphoprotein expression. For serum level comparison and phosphoprotein expression, two tumors were sliced, cultivated and analyzed visually. Scale bar: 200 μm.

**Figure S7.** Altered oncogenic signaling in prostate tumor slices. (A) pERK1/2 and pAKT(S473) expression, following IHC or immunoblotting analysis, in murine *Pten* loss-driven *in vivo* prostate tumors, freshly cut (0 h) and 24 h or 96 h cultured slices in the presence or absence of pAKT inhibitor (1 μM). Images are representative of two independent experiments. Scale bar: 50 μm. (B) Representative IHC analysis depicting p4EBP1 or pERK1/2 in human prostate tumor slices at 0 h or 24 h following culture onset. Data plots depict quantitated p4EBP1 and pERK1/2 (% area) in three sliced human prostate tumors (T1-T3) at 0 h or following 24 h slice culture. Scale bar: 500 μm. Two-tailed (paired) t-test was used for statistical comparison (p < *0.05).

**Figure S8.** Definition of minimally effective drug concentrations able to inhibit oncogenic signaling in tumor slices. (A) IHC of p4EBP1 in a freshly cut (0 h) KP AC slice, or after PI3K/mTOR inhibition by treatment with 0.1 μM or 1 μM dact, for 1h or 24 h. 1 μM, but not 0.1 μM, dact treatment effectively suppressed 4EBP1 phosphorylation at 24 h. Images are representative of two independent experiments. Scale bar: 1 mm. (B) IHC analysis of p4EBP1 and pAKT(S473) after 2 h treatment of KL ASC slices with 1 μM dact, or pERK1/2 after 2 h treatment of KL ASC slices with 0.5 μM sel, and comparative phosphoprotein expression in 0 h, 2 h, or 24 h DMSO-treated slices. Both compounds effectively inhibited their targeted pathways following 2 h of treatment. Images are representative of two independent experiments. Scale bar: 1 mm. (C) IHC depicting pAKT (S473), pSRC(Y416) in KL ASC or pERK1/2 in KL AC slices, following 24 h treatment with DMSO or 1 μM dact, 1 μM sar, or 0.5 μM sel. 20-40 tumors were analyzed per treatment experiment. All compounds effectively suppressed targeted signaling pathways following 24 h of treatment. While 1 μM dact treatment typically resulted in the partial inhibition of AKT phosphorylation (arrow points at residual signal), this fully inhibited 4EBP1 phosphorylation (shown in B). Scale bars: 1 mm. (D) IHC showing pSRC following 24 h treatment with 0.3 μM or 1 μM sar. 1 μM, but not 0.3 μM sar treatment effectively suppressed pSRC expression. Images are representative of four independent experiments. Scale bars: 500 μm.

**Figure S9.** Cytotoxic effects of short-term targeted therapy in ASC and AC slices. H&E images of matching DMSO-treated and drug-treated slices of ASC and AC showing necrotic cells and apoptotic bodies in treated slices (arrows). Scale bar: 50 μm.

**Figure S10.** Dact+sel combination treatment induces cytotoxicity, and this is not enhanced by sar addition. (A) Quantitation of Ki67 (%) in DMSO control (black) and neighboring drug-treated tumor slices (dact+sel in red; dact in blue; sel in grey). Representative IHC images depicting Ki67 in dact+sel-treated and matching DMSO control KL ASC or KP AC slices. Scale bar: 100 μm. (B) Cytotoxic responses shown as relative decrease in viability (%) following 24 h treatment of KL SCC of ASC, KL AC, and KP tumor slices with dact+sel or sar+dact+sel. Drug responses are measured as the ratio of quantitated viable tissue area in drug- and matching DMSO-treated slices. (C) Comparison of drug responses in the AC and SCC regions of KL ASC tissue slice samples following 24 h treatment with dact+sel. Drug responses are depicted as the relative decrease in viability (%), measured as the ratio of the quantitated viable tissue area in drug- and matching DMSO-treated slices. The tumor sample indicated by an arrow shows increased drug response in the AC region compared with the SCC region; H&E images depict masked dead (pink) and viable (purple) areas. The yellow solid line (H&E) and dotted lines (IHC) outline the SCC area that shows decreased drug response and lower pERK compared with the AC area marked by red lines. Scale bars: 500 μm (H&E), 1 mm (IHC).
