# Supplementary material for: Spatial aspects of oncogenic signalling determine the response to combination therapy in slice explants from Kras‐driven lung tumours
Source: J Pathol. 2018 Apr 2;245(1):101–13. doi: 10.1002/path.5059 (PMC5947161; doi:10.1002/path.5059)
Supplement: Supplementary file 13 — Table S1. Slice culture conditions and methods for different tumor pathologies [file PATH-245-101-s013.docx]

**Table S1. Slice culture conditions and methods for different tumor pathologies.**

| **Tissue** | **Slice thickness** | **Culture method** | **Culture medium** |
| --- | --- | --- | --- |
| Murine  NSCLC | 200 μm | Rotating platform with titanium grids (Figure S2A) | F12 (Gibco) supplemented with 100 U/mL penicillin, 100 μg/mL streptomycin (Gibco), 2 mM Glutamax (Gibco), and 22 mM glucose (Sigma-Aldrich, St. Louis, MO, USA) and with or without 10% fetal bovine serum (Gibco) |
| Human NSCLC | 200 μm | Rotating platform with titanium grids (Figure S2A) | F12 (Gibco) supplemented with 100 U/mL penicillin, 100 μg/mL streptomycin (Gibco), 2 mM Glutamax (Gibco), and 22 mM glucose (Sigma-Aldrich) and with or without 10% fetal bovine serum (Gibco) |
| Murine prostate tumor | 300 μm | Stationary platform (air-liquid interphase) with Millicell Cell Culture inserts (Merck Millipore, PTFE, pore size 0.4µm, (#PICM01250) | DMEM-F12 (Gibco) supplemented with 2% dextran-coated charcoal (Gibco), 1% insulin-transferrin-selenium (Gibco), 0.01% bovine serum albumin (Boehringer Mannheim, Mannheim, Germany), 10 ng/ml epidermal growth factor (Sigma-Aldrich), 100U/ml penicillin -100mg/ml streptomycin (Lonza, Basel, Switzerland) |
| Human prostate tumor | 300 μm | Rotating platform with titanium grids (Figure S2) | RPMF-4A supplemented with growth factors and androgen analogue R1881 |
