# Supplementary material for: Spatial aspects of oncogenic signalling determine the response to combination therapy in slice explants from Kras‐driven lung tumours
Source: J Pathol. 2018 Apr 2;245(1):101–13. doi: 10.1002/path.5059 (PMC5947161; doi:10.1002/path.5059)
Supplement: Supplementary file 14 — Table S2. Antibody details and verification [file PATH-245-101-s014.docx]

| **Antibody** | **Reference** | **Dilution** | **Validation method** |
| --- | --- | --- | --- |
| anti-  p44/22  (Erk1/2) | CST 4370 | 1:1000*  1:500 ** | MEK inhibition in murine NSCLC slice; blocking peptide (from CST); positive reference tissue (embryonic lung day 14) |
| anti-pAKT (S473)* | CST 4058 | 1:1000 | PI3K/AKT inhibition (BEZ235) in murine NSCLC slice |
| anti-pAKT (S473)** | CST 4060 | 1:300 | PI3K/AKT inhibition (BEZ235) in murine NSCLC slice |
| anti-p4EBP1 | CST 2855 | 1:1000 | mTORC1 inhibition (BEZ235) in murine NSCLC slice |
| anti-pAMPK | CST 2535 | 1:100 | positive and negative reference tissues (*Kras;Lkb1* and *Kras;p53* driven mouse lung tumors) |
| anti-pSRC(Y416) | CST 2101 | 1:250 | SRC inhibition (Saracatinib) in murine NSCLC slice |
| anti-p63 | Ab 53039 | 1:8000 | positive reference tissue (*Kras;Lkb1* ASC tumor) |
| anti-NKX2.1 (TTF-1) | Ab 133638 | 1:4000 | positive reference tissue (*Kras* AC tumor) |
| anti-  E-cadherin | CST 3195 | 1:400 | positive reference tissue (murine epithelium) |
| anti-Ki-67 | Thermo Scientific  RM-9106-S0 | 1:500 | positive reference tissue (murine embryonic tissue, *Kras* lung tumors) |
| anti-γH2AX | MilliporeMABE205 | 1:5000 | Irradiation of murine lung tissue slice |
| anti-LKB1** | CST 13031 | 1:500 | Murine *Kras;p53* driven tumors as positive reference tissue |
| anti-p53 ** | DAKO  M7001 | 1:50 | Human normal lung tissue and lymph node as a negative control |

**Table S2. Primary antibodies used in the study.**

CST = Cell Signaling Technology, Danvers, MA, USA

Ab = Abcam, Cambridge, UK

* in mouse tissue; ** in human tissue.
